# Midterm Outcomes of Ultrasound-guided Local Treatment for Infrapatellar Saphenous Neuroma Following Total Knee Arthroplasty

**DOI:** 10.7759/cureus.6565

**Published:** 2020-01-04

**Authors:** Glenn G Shi, Douglas S Schultz, Joseph Whalen, Steven Clendenen, Benjamin Wilke

**Affiliations:** 1 Orthopedics, Mayo Clinic, Jacksonville, USA; 2 Anesthesiology and Perioperative Medicine, Mayo Clinic, Jacksonville, USA

**Keywords:** total knee arthroplasty, revision total knee arthroplasty, neuroma

## Abstract

Background: While total knee arthroplasty (TKA) is a reliable treatment for advanced knee arthritis, up to 19% of patients after TKA remain dissatisfied, especially with residual pain. A less known source of medial knee pain following TKA is infrapatellar saphenous neuroma. Ultrasound-guided local treatment with hydrodissection and corticosteroid injection is an effective short-term solution. Our primary aim was to evaluate the durability of local treatment by comparing numeric pain scores for medial knee pain after TKA at pretreatment, one month following treatment, and midterm follow-up. A secondary aim was to identify associations of patient characteristics with degree of change in numeric pain score.

Methods: Retrospective chart review was performed to identify patients who had symptomatic infrapatellar saphenous neuroma following TKA and were treated with ultrasound-guided local treatment by hydrodissection and corticosteroid injection between January 1, 2012, and January 1, 2016. Those with follow-up less than three years were excluded. Patients who were unable to return for midterm follow-up were called. Numeric pain scores for the medial knee were recorded. Patient demographics, medical history, revision TKA status, number of prior knee surgeries, narcotic use, psychiatric disorders, and current tobacco use were also collected.

Results: Of 32 identified patients, 29 (7 men, 22 women, median age 65.9 years) elected to participate in this study with a mean (SD) follow-up of 4.6 (0.8) years. The median (range) pretreatment pain score was 9 (5-10). After local treatment, the median (range) numeric pain score was significantly lower at both one-month and midterm follow-up (5; P<0.001). The initial response to treatment was durable given that the difference between one-month and midterm follow-up scores was not significant (P=0.47). Advanced age was associated with less overall pain relief from pretreatment to midterm follow-up, while female sex, history of fibromyalgia, and TKA revision prior to treatment were associated with worsening pain from one-month to midterm follow-up (P<0.05).

Conclusions: Patients who underwent ultrasound-guided local treatment with hydrodissection and corticosteroid injection for painful postoperative infrapatellar saphenous neuroma following TKA experienced significant numeric pain score reduction. Pain relief remained consistent from 1onemonth to midterm follow-up.

Level of Evidence: Level IV, Case Series

## Introduction

Total knee arthroplasty (TKA) is a reliable surgical option for patients with tricompartmental knee arthritis for whom nonsurgical treatment has failed. In the United States, the rise in annual rates of primary TKA is matched with an increase in revision TKA [1]. Common sources of continued pain include infection, loosening, instability, polyethylene wear, and persistent pain. A lesser known but previously described source of medial knee pain following TKA is infrapatellar saphenous neuroma [2,3]. This nerve is susceptible to injury during the TKA procedure from the anteromedial arthrotomy, retractor placement, and less commonly, the tourniquet [2].

Although selective surgical neuroma excision is a possible solution with 84% of patients reporting good-excellent outcomes, nonsurgical management with ultrasound-guided local treatment with hydrodissection combined with corticosteroid injection has also been shown to be an effective short-term solution [2,3]. The long-term outcomes of patients who have undergone local treatment, however, are currently unknown.

The primary aim of our study was to compare numeric pain scores for medial knee pain after TKA at pretreatment, one month following treatment, and midterm follow-up. The secondary aim was to identify associations of patient characteristics with degree of change in numeric pain score. 

## Materials and methods

In this retrospective chart review approved by the Institutional Review Board, we identified patients diagnosed with symptomatic infrapatellar saphenous neuromas following TKA who were then treated with ultrasound-guided hydrodissection and corticosteroid injection between January 1, 2012, and January 1, 2016. Through chart review, diagnosis of infrapatellar saphenous neuroma was confirmed with preprocedural criteria of chronic medial-sided knee pain, history of ipsilateral TKA, lack of radiculopathy or neuropathy, or other documented sources of pain following TKA, such as loosening, infection, or malalignment. All patients had Tinel’s test producing point tender medial knee discomfort. The ultrasound-guided treatment was performed as described by Clendenen et al., using a 12-4 MHz transducer and a portable ultrasound. The infrapatellar branch of the saphenous nerve was identified and confirmed using electrical stimulation [2]. A mixture of dexamethasone 4 mg and 5 mL of 1.0% ropivacaine or 0.5% bupivacaine was injected adjacent to the nerve in the fascial plane, completing the hydrodissection portion.

The most recent numeric pain score was recorded from the chart review of patients with a follow-up greater than three years. Those who were identified as candidates for our study but were unable to return for midterm follow-up were called. After obtaining consent, the current numeric pain score (0, no pain, to 10, worst imaginable pain) for medial knee was recorded. Patient demographics, medical history, revision TKA status, number of prior knee surgeries, narcotic use, psychiatric disorders, and current tobacco use were also collected. 

Continuous variables were summarized with the sample median and range. Categorical variables were summarized with number and percentage of patients. Comparisons of medial knee visual analog scale (VAS) pain score prior to treatment, at one month following treatment, and at midterm follow-up between three and six years after treatment were made using paired Wilcoxon signed-rank tests. Associations of patient characteristics with degree of change in medial knee VAS pain scores from pretreatment to midterm follow-up and from one-month follow-up to midterm follow-up were evaluated using Spearman’s correlation test (for continuous or ordinal characteristics) or a Wilcoxon rank-sum test (for categorical characteristics). No adjustment for multiple testing was made in these exploratory analyses, and P-values less than 0.05 were considered as statistically significant. All statistical tests were two sided. Statistical analyses were performed using R Statistical Software (version 3.2.3; R Foundation for Statistical Computing, Vienna, Austria).

## Results

**Figure 1:**
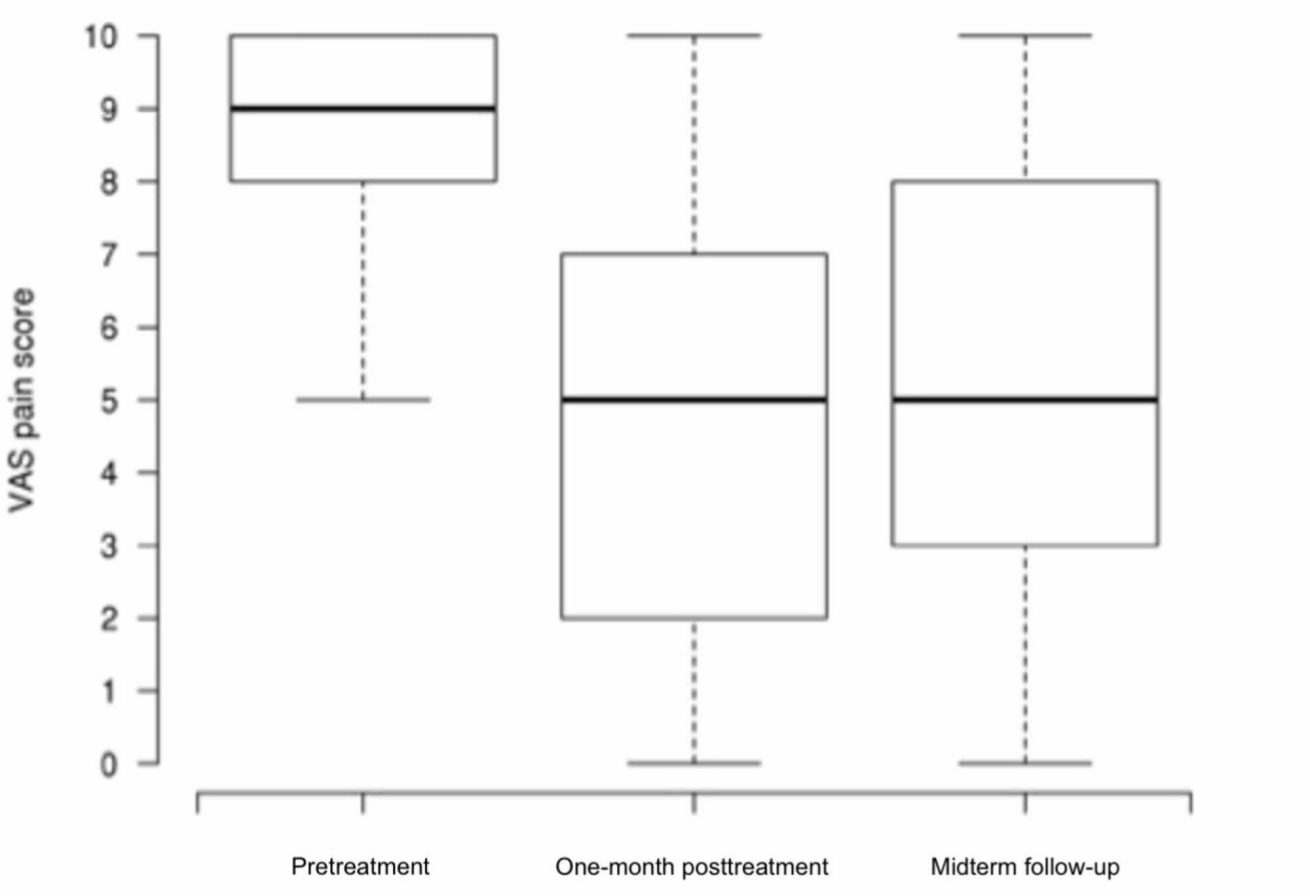
Boxplots Showing Medial Knee Numeric Pain Score Pretreatment, at One-month Follow-up, and at Midterm Follow-up at 4.6 Years VAS, visual analog scale.

Of 32 patients, 29 were identified (7 men, 22 women), with a median age of 65.9 (range: 34.7-79.8) years and a body mass index of 32.6 (range: 20.2-41.3), participating in this study with a mean (SD) follow-up of 4.6 years (range: 3-6) (Table 1). Six (21.4%) patients had a revised TKA prior to treatment, and the median number of previous knee surgeries was 2 (range: 1-11). Median pretreatment pain score was 9 (range: 5-10). After local treatment, the numeric pain score was significantly lower at both one-month and midterm follow-up (5, range: 0-10; P<0.001). The initial response to treatment was durable given that the difference between one-month and midterm follow-up scores was not significant (P=0.47, Figure 1).

**Table 1:** Patient Characteristics and Outcomes BMI, body mass index; TKA, total knee arthroplasty. ^a^Information given as no. (%) unless otherwise indicated. ^b^Information was unavailable regarding BMI (n=3), diabetes mellitus (n=1), revised TKA (n=1), history of psychiatric disorders (n=1), and history of tobacco use (n=1).

Variable^a^	Summary (N=29)
Pretreatment and treatment characteristics	
Age at treatment, median (range), years	65.9 (34.7 to 79.8)
Sex, male	7 (24.1%)
BMI, median (range)^b^	32.6 (20.2 to 41.3)
Diabetes mellitus^b^	5 (17.9%)
Fibromyalgia	10 (34.5%)
Revised TKA prior to treatment^b^	6 (21.4%)
Number of knee surgeries prior to treatment	
1	9 (31.0%)
2	8 (27.6%)
3	2 (6.9%)
4	3 (10.3%)
5	2 (6.9%)
>5	5 (17.2%)
Pretreatment narcotic use	6 (20.7%)
History of psychiatric disorders^b^	5 (17.9%)
History of tobacco use^b^	9 (32.1%)
Medial knee numeric pain score, median (range)	9 (5 to 10)
Treated knee	
Left	10 (34.5%)
Right	19 (65.5%)
Medial knee numeric pain score outcomes, median (range)	
One-month follow-up	5 (0 to 10)
Midterm follow-up (4.6 years)	5 (0 to 10)
One-month follow-up minus pretreatment	−4 (−8 to 0)
Midterm follow-up minus pretreatment	−3 (−10 to 1)
Midterm follow-up minus onemonth follow-up	0 (−8 to 7)

The associations of pretreatment characteristics with treatment outcome measured with the numeric pain score are shown in Table 2. Advanced age was associated with less overall pain relief from pretreatment to midterm follow-up (Table 2), while female sex, history of fibromyalgia, and TKA revision prior to treatment were associated with worsening pain from one-month to midterm follow-up (P<0.05) (Table 3).

**Table 2:** Associations of Continuous Patient Characteristics with Change in Numeric Pain Score from Pretreatment and One-month Follow-up to Midterm Follow-up at 4.6 years BMI, body mass index. ^a^Calculated as the medial knee numeric pain score at midterm follow-up following treatment minus the medial knee numeric pain score at pretreatment. ^b^Calculated as the medial knee numeric pain score at midterm follow-up following treatment minus the medial knee numeric pain score at one month following treatment. ^c^P-values result from Spearman rank correlation.

	Change From Pretreatment to Midterm Follow-up^a^		Change From One-month to Midterm Follow-up^b^	
Variable	Spearman r	P-Value^c^	Spearman r	P-Value^c^
Age at treatment	0.40	0.03	−0.02	0.91
BMI	0.00	0.99	−0.11	0.59
No. of knee surgeries prior to treatment	0.04	0.83	0.16	0.41
Pretreatment medial knee numeric pain score	−0.20	0.31	−0.01	0.95

**Table 3:** Associations of Categorical Patient Characteristics with Change in Numeric Pain Score from Pretreatment and One-month Follow-up to Midterm Follow-up at 4.6 Years TKA, total knee arthroplasty. ^a^This was calculated as the medial knee numeric pain score at midterm follow-up following treatment minus the medial knee numeric pain score at pretreatment. ^b^This was calculated as the medial knee numeric pain score at midterm follow-up following treatment minus the medial knee numeric pain score at one month following treatment. ^c^P-values result from a Wilcoxon rank-sum test.

	Change From Pretreatment to Midterm Follow-up^a^		Change From One-month to Midterm Follow-up^b^	
Variable	Median (Range)	P-Value^c^	Median (Range)	P-Value^c^
Sex		0.50		0.03
Male (n=7)	−2 (−10 to 0)		−1 (−8 to 1)	
Female (n=22)	−3 (−10 to 1)		0 (−5 to 7)	
Treated knee		0.35		0.78
Right (n=19)	−2 (−10 to 1)		0 (−8 to 6)	
Left (n=10)	−4 (−8 to 0)		0 (−2 to 7)	
Diabetes mellitus		0.48		0.56
Yes (n=5)	−4 (−10 to 0)		0 (−5 to 7)	
No (n=23)	−2 (−10 to 1)		0 (−8 to 6)	
Fibromyalgia		0.98		0.01
Yes (n=10)	−3 (−8 to 1)		2 (0 to 7)	
No (n=19)	−3 (−10 to 0)		0 (−8 to 5)	
Revised TKA—chart review		0.12		0.02
Yes (n=6)	−1 (−8 to 1)		2 (0 to 7)	
No (n=22)	−3 (−10 to 0)		0 (−8 to 5)	
Pretreatment narcotic use		0.72		0.66
Yes (n=6)	−3 (−10 to 0)		0 (−5 to 2)	
No (n=23)	−3 (−10 to 1)		0 (−8 to 7)	
History of psychiatric disorders		0.50		0.64
Yes (n=5)	−5 (−8 to 0)		0 (0 to 7)	
No (n=23)	−2 (−10 to 1)		0 (−8 to 6)	
History of tobacco use		0.75		0.24
Yes (n=9)	−2 (−8 to 0)		0 (−2 to 5)	
No (n=19)	−3 (−10 to 1)		0 (−8 to 7)	

## Discussion

Recalcitrant pain following TKA without infection or mechanical failure is a difficult clinical problem. Revision TKA is costly and ineffective if no clear source of pain exists [4,5]. A careful clinical evaluation can lead to diagnosis of periarticular neuroma, especially when common etiology has been ruled out. 

The infrapatellar branch of the saphenous nerve is a pure sensory nerve that provides cutaneous innervation to the anterior knee. Acute injury to this nerve can cause paresthesia and pain. We suspect the nerve may be injured at several points during surgery, including during tourniquet use, retraction, and medial parapatellar arthrotomy commonly used for anterior approach to the knee during arthroplasty. Direct iatrogenic trauma to the infrapatellar saphenous nerve during knee surgery is thought to be common given the complex and variable distribution of the terminal branches [6].

Although no intraoperative documentation of transection of the infrapatellar saphenous nerve exists upon chart review, current literature suggests the possibility of neural injury given that 55% to 100% of patients experience anterior knee paresthesia following TKA [7]. Symptoms of neural injury at this location commonly include point tender pain locations, paresthesia, and occasional electrical radiation of shock proximally with the Tinel sign [8]. Peripheral nerves tend to regenerate naturally following surgery; however, uncontrolled aberrant growth leads to formation of neuroma versus traction neuralgia. Chronic knee pain after TKA leads to morbidity, low functional status, arthrofibrosis, and measurable socioeconomic effect. 

Infrapatellar saphenous neuroma has been previously described as a source of persistent medial knee pain after an otherwise successful TKA [3]. Additionally, such neuropathic pain has been described to lead to possible chronic regional pain syndrome [9]. Noninvasive interventions have had limited success with neurogenic pain in animal study [10]. While there are alternative treatment options, including neuroma excision and cryoanalgesia, ultrasound-guided local treatment with hydrodissection and corticosteroid injection has proven to be effective in the short term [2,3,8,11]. However, midterm and long-term outcomes of the treatment are unknown. 

Our patients demonstrated hyperalgesia along the course of the infrapatellar nerve, which limited their ability to participate in walking and strengthening in postoperative physical therapy due to pain. All patients referred to the Pain Clinic had delays in diagnosis of over six months with majority of referrals from outside sources reported ongoing pain of one to two years following surgery. Delays or failure to recognize this entity can lead to long-term consequences. Buvanendran et al. showed that acute postoperative pain is a predictor of patients transitioning to chronic pain [12]. They found that anxiety, pain catastrophizing, depression, and limited physical mobility were factors contributing to the development of chronic pain [12]. In a study of more than 30,000 patients, Lewis et al. reported catastrophizing and preoperative pain as major factors for persistent postsurgical pain [13]. 

Although mean numeric pain score reduction reached significance in our study, several factors may have affected outcome after treatment. First, the diagnosis of neuroma was made clinically with history, examination characteristics of isolated point tender pain location, paresthesia, and Tinel sign. Not all patients routinely had objective nerve conduction velocity or electromyography prior to their local treatment to rule out other sources of referred pain, such as radiculopathy. The authors tried to address this by first performing ultrasound-guided diagnostic injection with local anesthetic to ensure that the patient had some adequate pain relief prior to proceeding with hydrodissection and corticosteroid injection. Second, several smaller branches may exist along the path of the infrapatellar branch of the saphenous nerve. By potentially not addressing all branches of the nerve, some neuromas may not have been treated, leaving patients with incomplete relief. Third, the saphenous nerve can become trapped at the level of the adductor canal, mimicking the medial knee pain seen with saphenous nerve neuroma [14]. Fourth, some patients may not have had a true neuroma but rather had entrapment of the nerve by the postoperative adhesions in the fascial planes producing a similar pain. Finally, some patients reported continued knee stiffness, despite substantial improvement in their numeric pain scale after treatment. We theorize that this stiffness is due to arthrofibrosis which may have developed secondary to pain in the first several months after TKA. Those patients dissatisfied with this stiffness may benefit from physical therapy or revision TKA [15].

The main limitations of our study are the retrospective design and relatively limited sample size. Due to the small sample size, power to detect associations is low, and therefore, the possibility of a type II error (i.e., a false-negative finding) is important to consider. Additionally, due to the retrospective design, there were no control group in our study.

## Conclusions

Ultrasound-guided local treatment with hydrodissection and corticosteroid injection is a safe and effective minimally invasive option for painful infrapatellar saphenous neuroma following TKA. Patients in this case series experienced significant numeric pain score reduction after hydrodissection and corticosteroid injection. Pain relief remained consistent from one-month to midterm follow-up. 
